# Camp Diarrhœa

**Published:** 1862-08

**Authors:** T. H. Walker

**Affiliations:** Chicago


### CAMP DIARRHŒA.

By T. H. WALKER, M. D., Chicago.

While I was engaged in the army before Corinth, diarrhœa prevailed as an epidemic throughout the camp, rendering the soldiers unfit for duty. I had a large number of patients under my charge, the majority of the cases being complicated with Intermittent Fever, producing great prostration and difficulty in treatment.

The disease was usually ushered in by loss of appetite, irritability of the stomach, frequent vomiting, and intermittent pulse. The evacuations from the bowels were very frequent —often as many as thirty in the day.

The treatment adopted by the Surgeons was very diverse and unsatisfactory.

By close examination, I found that the alimentary canal was loaded with undissolved ingesta. There was pain arising from the irregular and violent peristaltic action, with soreness throughout the course of the large intestine, the frequent stools accompanied by distressing tenesmus. The secretion from the liver was irregular, often excessive and of an acrid nature. The skin was sallow and the tongue heavily furred; the pulse compressed; frequently headache and disturbed sleep.

The causes are various, the hepatic disorder apparently often secondary to the intestinal irritation—apt to be provoked by excessive use of stimulants. Exposure to cold and wet, especially in the fall months. In this case there is usually less pain, unless it passes into dysenteric diarrhœa from the presence of aggravating influences.

The primary step in treatment is to remove the cause if it can be ascertained. My attention was first directed to the sanitary condition of the camp. I caused the tents to be rolled up, aired and dried, and all offensive matters to be removed; and both the invalided and healthy soldiers to be washed, and their clothes to be changed and cleansed as often as the conveniences of the camp would admit.

Next I looked to the food and cookery department, which I found extremely faulty, soldiers often neglecting to more than half cook their rations, with resulting indigestion and passage of the crude mass into the intestines, exciting and intensifying the morbid action. There was not much choice in diet to be had in camp, but by proper attention to its preparation a great barrier to the return of health was surmounted. The means for making a nourishing and suitable soup are always at hand in camp: meat, beans, rice, and even potatoes, which, by the skill of any competent cook, can easily be made into a soup perfectly harmless and wholesome.

So far as medication was concerned, I usually commenced my treatment by giving large doses of Sulphate of Magnesia to wash out the contents of the bowels. I then administered Ipecac, Opium, Hydrarg. Cum Creta and Quinine, the proportion of each varying with the peculiarities of each case. In the course of twenty-four hours the evacuations would assume a dark color, and convalescence rapidly ensue. The Ipecac and Opium were given in moderate doses. The Hydrarg. Cum Creta appeared to act in the most salutary manner in cases where Calomel would irritate excessively. The Quinine, when there was intermittent complication, when given alone did not fail to aggravate the difficulty, but in the above combination had a most happy effect.

Of eighty-eight cases under treatment at one time, the whole were reported fit for duty in seven days from the commencement of the treatment.

The Brigade Surgeon, who visited the camps with reference to the presence of this scourge, was so strikingly impressed by the marked superiority of this treatment to that relied upon by others, that he gave it cordial and high approval and recommended it to the other Surgeons. Previously too much reliance had been placed on Calomel, Opium and astringents, and, worst of all, upon alcoholic stimulants; but since then Sulphate of Magnesia takes the first rank. One hundred and fifty barr[illegible] of it were ordered for Gen. Grant's division by the Medical Director, to be used as above indicated.

The writer, in concluding this hasty sketch of the method relied upon by him in opposing this "scourge of the camp," begs leave to say that while he does not claim any novelty in it, or new discovery, yet the application never has, to his knowledge, previously received the high place in the confidence and practice of the Army Surgeons which the experience above recorded showed it ought to receive.

July 30th, 1862.

